# Reconstruction of Aggressive Grade 3 Calcaneal Giant Cell Tumour with Femoral Head Allograft: A Case Report

**DOI:** 10.5704/MOJ.2603.020

**Published:** 2026-03

**Authors:** M Ibrahim, Z Khan

**Affiliations:** Department of Trauma and Orthopedic Surgery, Rehman Medical Institute, Peshawar, Pakistan

**Keywords:** giant cell tumours, calcaneus, bone neoplasms, limb salvage, allograft

## Abstract

Giant Cell Tumour (GCT) of bone is a benign, locally aggressive neoplasm. GCT of the foot is rare, comprising of about 5% of cases of all GCTs. GCT of Calcaneus is exceedingly rare, comprising of 1.2% of all calcaneal tumours. Due to its uncommon occurrence at this site, diagnosis can be delayed. In this report, we present the case of a Campanacci Grade 3 GCT of calcaneus in a 43-year-old female patient with 8 months history of worsening pain and disability. We treated her successfully by resection of Os-calcis and reconstruction with a femoral head allograft and K-wire fixation, a relatively cheaper and technically lesser challenging method of reconstruction. Three years’ post-surgery, she remains disease free, and her graft has healed. She continues to walk independently and remains disease free clinically and radiologically. We discuss a comparison with other reported cases where surgeons have opted for detailed intra-lesional curettage (DILC) and cementoplasty to fill the defect for a Grade 2 disease, some have even used a sural for soft tissue coverage with a maximum follow-up of two years. While in our patient we went for Calcanues resection and reconstruction with allograft because of a Grade 3 disease that poses greater risk of local recurrence with just DILC. Our patient has a three-year follow-up where she remains disease free.

## INTRODUCTION

Giant Cell Tumour (GCT) of bone is a benign, locally aggressive neoplasm commonly involving the appendicular skeleton in the epiphyseal, juxta-articular regions accounting for approximately 3–5% of all primary bone tumours. It predominantly affects individuals in their third and fourth decades of life, with a slight predilection for females.

The prevalence of tumours in the foot and ankle region is rare^1^, with the complex anatomy of this region making interventions challenging for the surgeons. Of all the osseous tumours in the skeleton, the incidence of the tumours of the foot comprises only 3%, of which one-third reside in the calcaneus. GCT of the foot is rare, comprising of about 5% of cases of all GCTs. GCT of the calcaneus, however, is exceedingly rare, comprising of only about 1.2% of all calcaneal tumours^2^. Clinically, patients can present with nonspecific symptoms such as persistent painful swelling which gradually worsens over time. These symptoms can lead to significant functional impairment, difficulty in weight-bearing, joint deformity, permanent disability or even a pathological fracture.

Plain radiographs serve as the initial diagnostic modality, typically revealing a lytic expansile lesion with or without a cortical rim breach. Magnetic Resonance Imaging (MRI) and Computed Tomography (CT) scans are essential for local staging, cross sectional assessment of the bony destruction and the associated soft tissue involvement in advanced disease. These imaging techniques are also required for surgical planning and Campanacci grading. A core needle biopsy confirms the diagnosis which is essential as some neoplastic lesions have similar radiological features which may be misleading especially when complicated by a pathological facture.

Management of GCTs predominantly require surgical treatment where the main goal of the treatment is to remove the tumour completely while preserving the local anatomy, restoring function and minimising local recurrence. The standard procedure offered is DILC for Grade 1 or 2 disease, while resection plus reconstruction is offered for Grade 3 disease which has structural compromise. Similarly, in foot or calcaneum, if the patient has a Grade 1 or 2 lesion with intact cortices an attempt to salvage the involved bone is preferred with DILC, the remaining cavity can be filled with polymethyl methacrylate (PMMA). In a Grade 3 lesion, however, due to the structural compromise of the involved bone DILC becomes difficult and has higher recurrence rates^3^. Thus, in such cases, resecting the tumour in-toto is preferred with reconstruction of the remaining defect with bone grafts, including autografts and allografts, and prosthesis to restore anatomical integrity and function^4^.

## CASE REPORT

A 43-year-old female with no significant past medical history presented with an eight-month history of progressively worsening left heel pain and swelling. She was initially treated as plantar fasciitis but then reported continued difficulty in weight bearing on the affected side, leading to significant functional impairment. She denied any history of trauma or injury to and there were no preceding symptoms suggestive of infection or systemic illness.

**Fig. 1:**
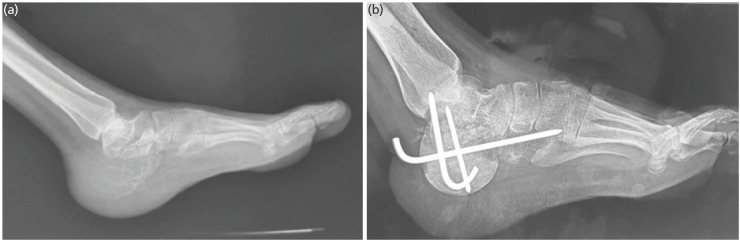
a) Lateral view of the left foot showing an expansile lytic lesion of the calcaneum with no visible cortical margin posteriorly; Campanacci Grade 3. (b) Post-operative radiograph, lateral view, at two weeks after removal of stitches, with femoral head allograft implanted after resection and fixation with Kirschner.

On presentation to our sarcoma service, there was a noticeable swelling with normal overlying skin and painful range of motion at ankle and sub-talar joint. A plain radiograph of the left foot revealed an expansile lytic lesion involving the entire calcaneus with significant cortical breech consistent with a Campanacci’s Grade 3 disease (Fig. 1a).

**Fig. 2:**
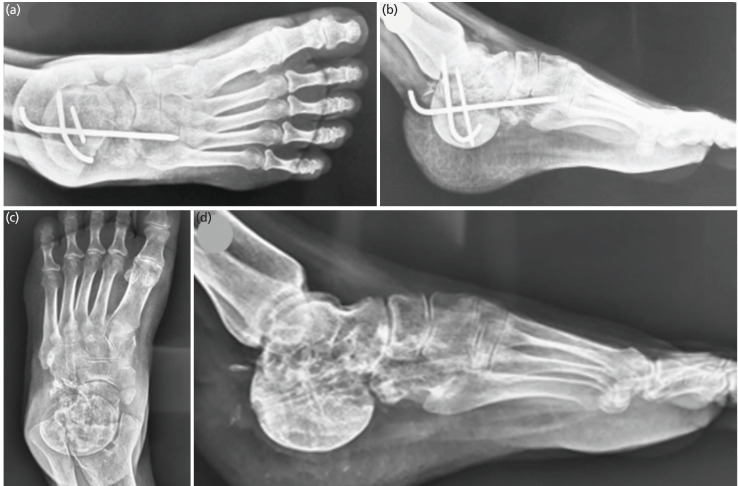
(a and b) Follow-up radiographs at three months showing early radiological signs of graft incorporation. (c and d) The 36 months follow-up radiographs (AP lateral radiographs), showing complete incorporation of allograft, no local recurrence and resorption, with some alteration in calcaneal pitch angle.

**Fig. 3:**
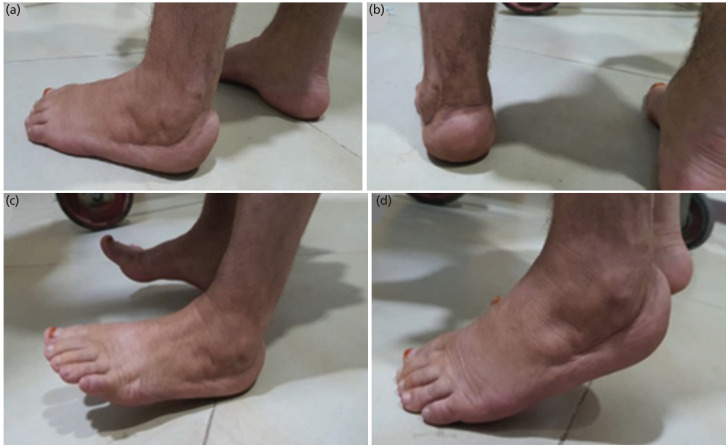
Clinical photograph at 36 months’ post-surgery, (a and b) showing a mature scar with plantigrade foot, and (c and d) patient being able to stand on heels and tip toe.

A core needle biopsy performed under local anaesthesia confirmed the diagnosis of Giant Cell Tumour (GCT) of the bone. Considering the local extent of disease, a wide local excision of the Os-calcis through a lateral approach was performed under general anaesthesia and antibiotics cover, with preservation of neurovascular bundles, peronei and medial structures. The resultant defect was reconstructed with a fresh frozen femoral head allograft and fused with K wires. The Tendo achilles was tenodesed into the femoral head allograft in neutral foot position with the K wires and a back slab in the neutral dorsiflexion position was applied post-operatively (Fig. 1b). The patient's post-operative recovery was uneventful and was kept non-weight bearing for three months in a plaster. At three months plaster was removed following early radiological signs of graft incorporation and protected weight bearing in a walking boot was commenced (Fig. 2a and 2b). She was followed serially every three months in the outpatient’s department with clinical and radiological assessment. She was fully weight bearing and disease free by 18 months at which point the K wires were removed following radiological evidence of complete graft incorporation (Fig. 2c and 2d). At her last follow-up, three years’ post-surgery, she continues to walk independently and remains disease free with a foot and Ankle disability index score of 83.1 and American Orthopaedic Foot and Ankle Society (AOFAS) score of 69 (Fig. 3).

## DISCUSSION

Giant cell tumours (GCTs) of bone are relatively uncommon, accounting for about 3–5% of all primary bone tumours^2^. They most commonly occur in the epiphyseal region of long bones however the axial skeleton can be involved infrequently as well, with GCTs in the calcaneum being exceedingly rare.

Treating GCTs in the heel presents unique challenges due to the complex anatomy of the area. Detailed or extended intra-lesional curettage with preservation of bone stock and function is the mainstay of treatment for all Grade 1 and 2 lesions and some Grade 3 lesions with resultant cavity filled with PMMA or cancellous bone graft (Table I)^1-5^. Many authors in the table below have opted for detailed intra-lesional curettage for their calcaneal GCTs. Because in a Grade 2 disease the tumour is intra-cortical and has not breached the cortex. In this situation, the involved bone of the foot can be salvaged, and the remaining defect’s structural integrity can be restored with PMMA. However, Grade 3 lesions have a reported higher risk of local recurrence (up-to 70%) due to their soft tissue involvement. A curettage would not be able to achieve the primary goal of removing the whole tumour in a way to keep the recurrence chances minimal. In such cases, one may consider wide excision to reduce this risk but at the cost of more morbid surgery and reduced function.

**Table I:** Case reports of different reported methods of resection and reconstruction methods used for the calcaneus on the basis of Campanacci grading.

**Author**	**Publication year**	**Campanacci Grade (1-3)**	**Surgical technique**	**Follow-up (Months)**	**Clinical Outcome (At last follow-up)**	**Functional Outcome (At last follow-up)**
Dhillon MS, *et al*^1^	2007	Grade 3 (Multicentric GCT)	DILC + Cementoplasty	18	No local recurrence, patient developed GCT in the ipsilateral 3rd toe.	Symptom free
Kamal AF, *et al*^3^	2016	Grade 3	WLE + Femoral head allograft and Sural flap (soft tissue coverage)	12	No recurrence	Symptom free
Batheja D, *et al*^2^	2020	Grade 2	DILC + Cementoplasty	24	No recurrence	Symptom free
Wiratnaya IGE, *et al*^5^	2022	Grade 2	DILC	9	No recurrence	Symptom free
Kamal AF, *et al*^4^	2022	Grade 2	DILC + Cementoplasty + Femoral head allograft.	19	No recurrence	Symptom free
Zeeshan *et al* (our Case)		Grade 3	WLE + reconstruction with femoral head allograft	36	No recurrence	Symptom free, mobile independently.

Following resection of Os-calcis, various reconstruction techniques can be used including bone graft, autografts and allografts, and 3D printed implants^4^. Autografts can be in the form of vascularised iliac crest or fibula graft, which requires microsurgical skills. Customised 3D printed implants offer a more trendy choice but it comes with a larger financial tag and is further compromised by its non-availability in financially constrained health care systems. To overcome these challenges, we opted for a femoral head allograft to fill the defect which due to its hemi-spherical shape, offers the best fit for the defect in the calcaneus.

Various studies have reported satisfactory long-term survivorship of allografts, if they survive an infection in the first 12 months and graft resorption or fracture at 36 months (Table I). The success of allografts is often measured not only by survival rates but also by functional outcomes, with many patients achieving satisfactory results that allow for significant functional recovery. To our knowledge, femoral head used purely as an allograft to reconstruct the calcaneal defect has been reported only once in the English literature.

In conclusion, GCT is a rare tumour and its presentation in calcaneum is even more rarer. Early presentation and diagnosis are critical, as timely intervention can prevent the need for more aggressive procedures, such as calcanectomy or even amputation. This case highlights the importance of considering a cheaper, technically lesser challenging and relatively easily available option for reconstruction of Os-calcis following its wide excision in neoplastic cases.

## CONFLICT OF INTEREST

The authors declare no potential conflict of interest.
